# Adsorption of Favipiravir on pristine graphene nanosheets as a drug delivery system: a DFT study†

**DOI:** 10.1039/d3ra03227b

**Published:** 2023-06-09

**Authors:** Mahmoud A. A. Ibrahim, Manar H. A. Hamad, Amna H. M. Mahmoud, Gamal A. H. Mekhemer, Peter A. Sidhom, Shaban R. M. Sayed, Nayra A. M. Moussa, Abdallah I. M. Rabee, Eslam Dabbish, Tamer Shoeib

**Affiliations:** a Computational Chemistry Laboratory, Chemistry Department, Faculty of Science, Minia University Minia 61519 Egypt m.ibrahim@compchem.net; b School of Health Sciences, University of KwaZulu-Natal Westville Campus Durban 4000 South Africa; c Department of Pharmaceutical Chemistry, Faculty of Pharmacy, Tanta University Tanta 31527 Egypt; d Department of Botany and Microbiology, College of Science, King Saud University P.O. Box 2455 Riyadh 11451 Saudi Arabia; e Leibniz-Institut für Katalyse Albert-Einstein-Str. 29 A 18059 Rostock Germany; f Department of Chemistry, The American University in Cairo New Cairo 11835 Egypt

## Abstract

The efficiency of pristine graphene (GN) in the delivery process of the Favipiravir (FPV) anti-COVID-19 drug was herein revealed within the FPV⋯GN complexes in perpendicular and parallel configurations in terms of the density functional theory (DFT) method. Adsorption energy findings unveiled that the parallel configuration of FPV⋯GN complexes showed higher desirability than the perpendicular one, giving adsorption energy up to −15.95 kcal mol^−1^. This favorability could be interpreted as a consequence of the contribution of π–π stacking to the overall strength of the adsorption process in the parallel configuration. Frontier molecular orbitals (FMO) findings demonstrated the ability of the GN nanosheet to adsorb the FPV drug by the alteration in the *E*_HOMO_, *E*_LUMO_, and *E*_gap_ values before and after the adsorption process. Based on Bader charge results, the FPV drug and GN sheet exhibited electron-donating and -accepting characters, respectively, which was confirmed by the negative sign of the computed charge transfer (*Q*_t_) values. The FPV(R)⋯T@GN complex showed the most desirable *Q*_t_ value of −0.0377*e*, which was in synoptic with the adsorption energy pattern. Electronic properties of GN were also altered after the adsorption of the FPV drug in both configurations, with more observable changes in the parallel one. Interestingly, the Dirac point of the GN sheet coincided with the Fermi level after the adsorption process, indicating that the adsorption process unaffected the presence of the Dirac point. The occurrence of the adsorption process was also noticed by the existence of new bands and peaks in the band structure and DOS plots, respectively. Short recovery time rendered the GN nanosheet an efficient FPV drug delivery system. The obtained findings provide new insight into the biomedical applications of the GN sheet as a promising drug delivery system.

## Abstract 2

The efficacy of the graphene nanosheet to adsorb Favipiravir drug candidate was investigated at various adsorption sites using DFT methods.

## Graphical abstract

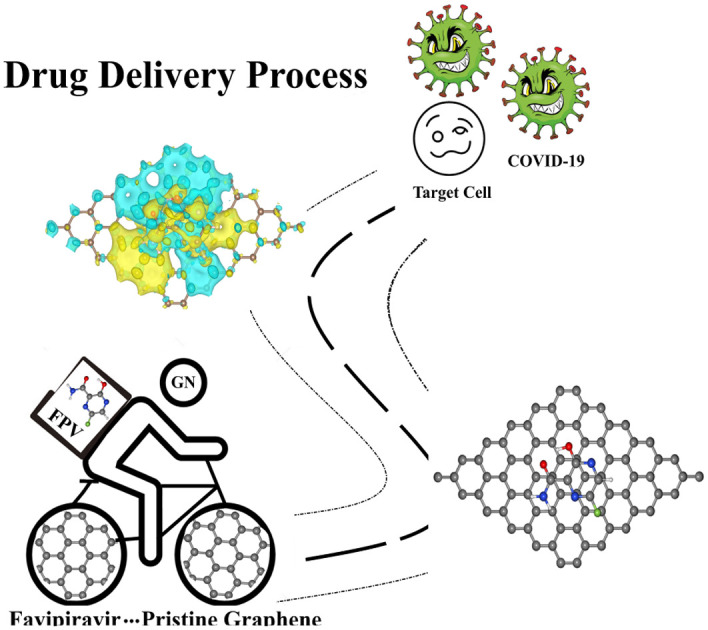

## Introduction

Two-dimensional (2D) nanomaterials have seized an exceeding interest by dint of their privileged features, such as specific surface area, transparency, and mechanical properties.^1,2^ Pristine graphene (GN), a premier discovered 2D material, was previously characterized by its large surface area, robust charge carrier mobility, exquisite thermal conductivity, lower toxicity, and mechanical and electronic properties.^3–8^ GN sheet and GN-based materials have been used in many applications, such as transparent electrodes, energy storage devices, photodetectors, and sensors.^9–18^ The GN sheet has also demonstrated its efficiency in biomedical applications, such as cancer therapeutics, imaging, diagnosis, and biosensors.^19–25^ Accordingly, tremendous interest has been directed toward using the GN-based material in drug/gene delivery and biomedical/tissue engineering.^21,22,26–29^ It was reported that the loading ratio of the GN-based material showed higher favorability compared with that of other drug delivery systems.^30,31^ As a point of departure for using GN as a delivery system, Liu *et al.* earlier reported the productive impact of GN-based material on delivering water-insoluble cancer drugs.^23^ Afterward, recent affirmations were announced for the superb amplitude of GN-based material as a promising antiviral drug delivery system.^32,33^ Nevertheless, a thorough literature review unraveled that the application of GN as a drug delivery system for antiviral drugs is still at an early stage.

Favipiravir (FPV), known as Avigan, was earlier utilized as an antiviral drug to treat diverse infections caused by the Ebola virus and new forms of influenza.^34–39^ More recently, FPV has been authorized to combat the COVID-19 pandemic.^40^ To enhance the safety and efficacy of the FPV drug, a plethora of nanostructures was developed for the FPV drug delivery process. However, the utilization of GN sheets in delivering the FPV drug is still ambiguous.

**Fig. 1:**
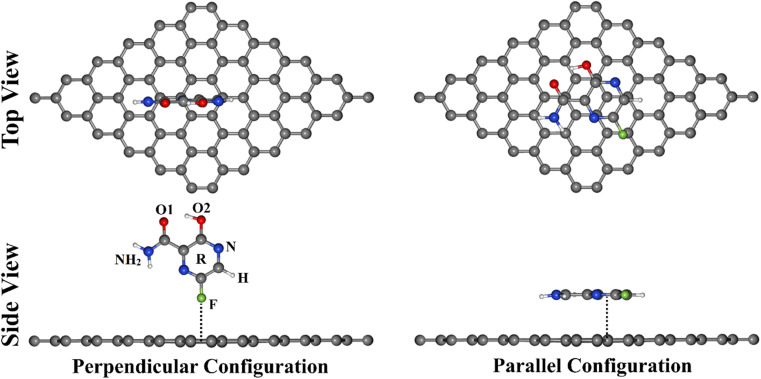
Top and side representations of the adsorption of FPV drug on GN sheet in perpendicular and parallel configurations. Active sites of FPV labeled: F atom (F), H atom (H), N atom (N), O atom (O), NH_2_ group (NH_2_), and the ring of FPV drug (R).

To provide an avenue for the biomedical application of the GN sheet as an FPV drug delivery system, various DFT calculations were employed to thoroughly investigate the adsorption process within the FPV⋯GN complexes in perpendicular and parallel configurations (Fig. 1). In that spirit, all plausible interacting sites of FPV drug were taken into account for adsorbing on the GN sheet toward identifying the most suitable structure of the FPV⋯GN complexes. Geometric structures and adsorption energy calculations were first performed for the investigated complexes. Frontier molecular orbital (FMO) calculations were then executed for the FPV drug and the GN sheet before and after the adsorption process. Upon the most stable complexes, detailed elucidation of the electronic properties was established by executing the density of state (DOS), band structure, and charge density analyses. As well, the recovery time was evaluated for the most stable complexes. The obtained results would provide an entire route for utilizing GN sheets in future biomedical applications as an antiviral drug delivery system.

### Computational methods

Density functional theory (DFT) method^41,42^ was implemented for all calculations of FPV drug adsorption on pristine GN *via* the Quantum ESPRESSO 6.4.1 package.^43,44^ The Perdew–Burke–Ernzerhof (PBE) exchange–correlation functional was applied to describe the electronic interactions within the Generalized Gradient Approximation (GGA).^45^ For describing the interaction of valence electrons and atomic cores, the ultrasoft pseudopotential (USPP) was employed.^46^ The dispersion interaction was corrected using the Grimme (DFT-D2) method.^47^ Additionally, the kinetic energy and charge density cutoffs in all computations were chosen, according to the examined values in Fig. S1,† to be 40 Ry and 400 Ry, respectively. The geometry optimization for all structures was performed at convergence criteria of 10^−5^ eV for energy and 10^−4^ eV Å^−1^ for force. The 4 × 4 × 1 and 8 × 8 × 1 *k*-points were used to simplify the first Brillouin zone based on Monkhorst–Pack grids for both geometric optimization and electronic structure calculations, respectively (Fig. S1†). To speed up the convergence, the Marzari–Vanderbilt smearing technique was utilized.^48^ To prevent the interactions between the periodic cells in the *z*-direction, a vacuum with a thickness of 20 Å was created along the vertical direction of the GN surface in all calculations.

The tendency of the GN sheet to adsorb the FPV drug was fully unveiled within the perpendicular and parallel configurations of the FPV⋯GN complexes (Fig. 1). A supercell of 6 × 6 × 1 was constructed to calculate the adsorption energies and involved 72 carbon atoms in the sheet. Adsorption energy (*E*_ads_) was calculated using the following equation:

1 *E*_ads_ = *E*_FPV⋯GN_ − (*E*_FPV_ + *E*_GN_)

where *E*_FPV⋯GN_, *E*_FPV_, and *E*_GN_ represent the energies of the complex, the adsorbed FPV drug, and the GN sheet, respectively. Frontier molecular orbitals (FMO) calculations were performed to further understand the adsorption process of the FPV drug on the GN sheet. By means of the FMO analysis, energies of the highest occupied molecular orbitals (*E*_HOMO_) and the lowest unoccupied molecular orbitals (*E*_LUMO_) were calculated. Afterwards, the energy gap (*E*_gap_) was computed as follows:

2 *E*_gap_ = *E*_LUMO_ − *E*_HOMO_

The charge density difference (Δ*ρ*) was estimated as illustrated in the following equation:

3 Δ*ρ* = *ρ*_FPV⋯GN_ − *ρ*_GN_ − *ρ*_FPV_

For generating Δ*ρ* maps, the Visualization for Electronic and Structural Analysis (VESTA) package was utilized.^49^ Bader charge method^50,51^ was devoted to assessing the charge transfer (*Q*_t_) to or from the GN sheet based on eqn (4).

4 *Q*_t_ = *Q*_combined GN_ − *Q*_isolated GN_

where *Q*_combined GN_ and *Q*_isolated GN_ are the total charge of the GN sheet after and before the adsorption process, respectively. A further understanding of the electronic properties of the GN sheet was established by executing the band structure and total and projected density of states (TDOS/PDOS) calculations. Recovery time (*τ*) was also computed to assess the difficulty of the desorption process for the studied complexes based on the following equation:

5 *τ* = *v*^−1^exp(−*E*_ads_/*KT*)

where *v*^−1^ sets for attempt frequency with a value of 10^12^ s^−1^. *K* stands for the Boltzmann constant. *T* refers to the temperature with a value of 298.15 K.

## Results and discussion

### Geometric structures

**Fig. 2:**
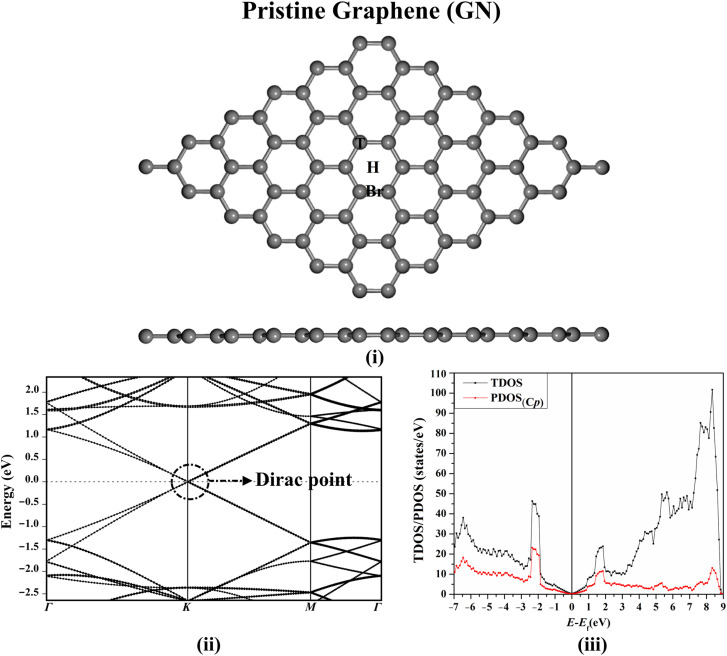
Views of (i) relaxed structure with all possible adsorption sites, (ii) electronic band structure, and (iii) DOS plots of the pure GN sheet. The Dirac point presents at the Fermi level at zero.

Prior to investigating the adsorption process of the FPV drug, the structure of the GN sheet was initially modeled and optimized for reaching the equilibrium geometry. The relaxed structure of the GN sheet, along with its electronic band structure and DOS plots, are presented in Fig. 2.

After the optimization of the GN sheet, the lattice constant was *a* = 2.47 Å, which was compatible with the preceding studies,^52,53^ as illustrated in Fig. S1.† According to Fig. 2(i), three adsorption sites were noticed on the GN sheet, represented as the top (T) site above the carbon atom, the bridge (Br) site at the midpoint of the carbon–carbon bond, and the hollow (H) site at the centre of the hexagonal ring of the sheet. According to Fig. 2(ii) and (iii), the electronic band structure and the DOS plots of the GN sheet demonstrated its semiconducting property, indicated by the presence of the Dirac point at the Fermi level.

### Adsorption energy calculations

**Fig. 3:**
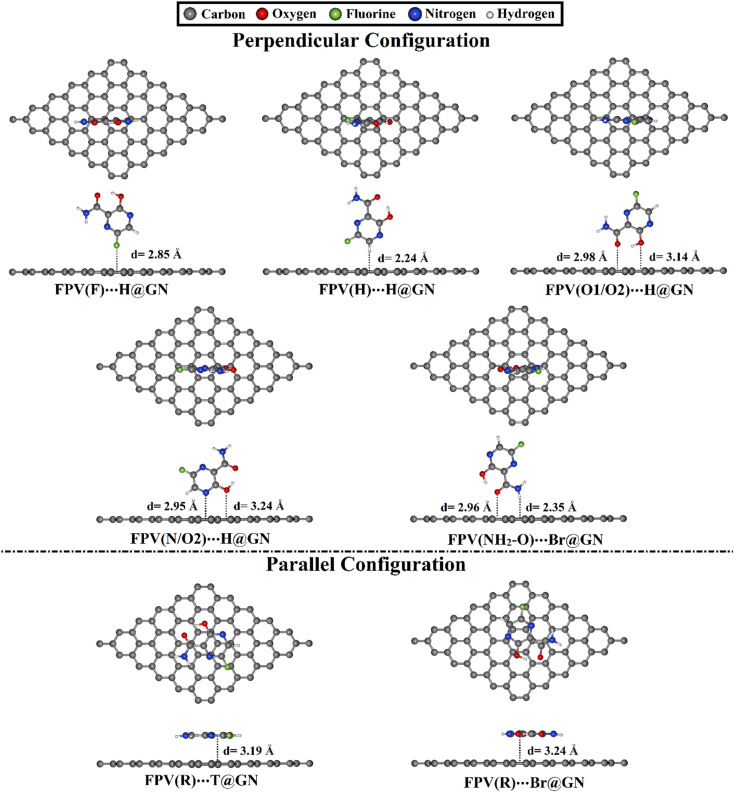
Relaxed structures of the most preferred FPV⋯GN complexes in perpendicular and parallel configurations with top and side views. Equilibrium distances (*d*) are provided in Å.

To deeply investigate the efficiency of the GN nanosheet as an FPV drug delivery system, all possible reactive sites of the FPV drug were subjected to specific adsorption sites with respect to the GN nanosheet in perpendicular and parallel configurations (see Fig. 1). All designed FPV⋯GN complexes were first subjected to relaxation (Fig. S2†). Looking at the relaxed FPV⋯GN complexes displayed in Fig. S2,† the investigated plausible reactive sites of the FPV exhibited potential versatility toward adsorbing on the surface of the GN nanosheet without any distortion into their rearrangement in the molecular structure of FPV. In order to comprehend the stability of the FPV⋯GN complexes, the adsorption energies of all relaxed complexes were computed, and the findings are compiled in Table 1. Upon the estimated adsorption energies, the most preferred FPV⋯GN complexes are presented in Fig. 3.

**Table 1:** Adsorption energy (*E*_ads_, kcal mol^−1^) and equilibrium distance (*d*, Å) of FPV⋯GN complexes, as well the charge transfer (*Q*_t_, e) of the GN sheet after the adsorption process

System	Adsorption site^a^	Bond	*d* (Å)	*E*_ads_ (kcal mol^−1^)	*Q*_t_ b (e)
**Perpendicular configuration** c					
FPV(F)⋯GN	T	F⋯GN	2.94	−3.32	−0.0070
	Br	F⋯GN	2.93	−3.34	−0.0074
	H	F⋯GN	2.85	−3.51	−0.0079
FPV(H)⋯GN	T	H⋯GN	2.44	−5.06	−0.0154
	Br	—d	—d	—d	—d
	H	H⋯GN	2.24	−5.83	−0.0170
FPV(O1/O2)⋯GN	T	O1⋯GN	3.06	−5.23	−0.0076
		O2⋯GN	3.11		
	Br	O1⋯GN	3.03	−5.40	−0.0078
		O2⋯GN	3.13		
	H	O1⋯GN	2.98	−5.48	−0.0099
		O2⋯GN	3.14		
FPV(N/O2)⋯GN	T	—d	—d	—d	—d
	Br	N⋯GN	3.03	−6.52	−0.0014
		O2⋯GN	3.38		
	H	N⋯GN	2.95	−6.83	−0.0035
		O2⋯GN	3.24		
FPV(NH_2_/O1)⋯GN	T	NH_2_⋯GN	2.29	−6.59	−0.0221
		O1⋯GN	3.06		
	Br	NH_2_⋯GN	2.35	−6.69	−0.0206
		O1⋯GN	2.96		
	H	NH_2_⋯GN	2.25	−6.62	−0.0151
		O1⋯GN	3.08		

**Parallel configuration** c					
FPV(R)⋯GN	T	R⋯GN	3.19	−15.95	−0.0377
	Br	R⋯GN	3.24	−15.20	−0.0322
	H	—d	—d	—d	—d

a All adsorption sites on the surface of GN sheet are shown in Fig. 2(i).

b *Q*_t_ was determined according to eqn (3).

c Perpendicular and parallel configurations for all relaxed FPV⋯GN complexes are presented in Fig. S2.

d No adsorption was detected within the designed configuration.

For all investigated configurations of FPV⋯GN complexes, the appreciable potentiality of the GN sheet for adsorbing the FPV drug was noticed and confirmed by the small values of equilibrium distances (Fig. S2†). For perpendicular configuration, the potency of the GN sheet to adsorb the FPV drug generally grew by increasing negative *E*_ads_ values according to the following pattern: FPV⋯T@GN < FPV⋯Br@GN < FPV⋯H@GN. For example, the *E*_ads_ values of the FPV(F)⋯T@GN, FPV(F)⋯Br@GN, and FPV(F)⋯H@GN complexes were −3.32, −3.43, and −3.51 kcal mol^−1^, respectively. With an exception, the FPV(NH_2_/O1) was favorably adsorbed at the Br@GN site, compared to FPV(NH_2_/O1)⋯T@GN and FPV(NH_2_/O1)⋯H@GN with *E*_ads_ values of −6.69, −6.59, and −6.62 kcal mol^−1^, respectively (see Table 1). Among all the FPV⋯GN complexes, the most preferred negative *E*_ads_ was observed for the FPV(N/O2)⋯H@GN complex in the perpendicular configuration with a value of −6.83 kcal mol^−1^. This observation could be ascribed to the contributions of the O2⋯GN interaction to the total adsorption energy of the FPV(N/O2)⋯H@GN complex.

For parallel configuration, the hexagonal ring (R) of the FPV drug was a suitable site for the adsorption process on the surface of the GN sheet. Notably, the FPV(R)⋯T@GN complex exhibited the largest negative *E*_ads_, followed by FPV(R)⋯Br@GN complex with *E*_ads_ values of −15.95 and −15.20 kcal mol^−1^, respectively. The abovementioned *E*_ads_ values ensured the significant contribution of π–π stacking.

To sum up, the H@GN site preferred to adsorb the FPV drug in a perpendicular configuration for all active sites except the (NH_2_/O1) active site, which preferred to be adsorbed at the Br@GN site. While in the parallel configuration, the most appropriate *E*_ads_ was ascribed to the FPV(R)⋯T@GN complex. Significantly, the adsorption process in parallel configuration for the FPV⋯GN complexes was more desirable than that of the perpendicular configuration.

### Frontier molecular orbitals (FMO) calculations

To deeply unveil the effect of the adsorption process on the electronic properties of the studied systems, the energies of the highest occupied molecular orbitals (*E*_HOMO_) and the lowest unoccupied molecular orbitals (*E*_LUMO_) along with the energy gap (*E*_gap_) were assessed. The FMO energetic values before and after the adsorption process are listed in Table 2.

**Table 2:** The energies of the highest occupied molecular orbitals (*E*_HOMO_, eV), the lowest unoccupied molecular orbitals (*E*_LUMO_, eV), along with the energy gap (*E*_gap_, eV) before and after the adsorption process within the relaxed FPV⋯GN complexes

System	Adsorption site^a^	*E*_HOMO_ (eV)	*E*_LUMO_ (eV)	*E*_gap_ b (eV)
GN sheet		−2.356	−2.337	0.0185
FPV drug		−6.078	−3.168	2.9094

**Perpendicular configuration** c				
FPV(F)⋯GN	T	−2.081	−2.065	0.0155
	Br	−2.080	−2.065	0.0155
	H	−2.080	−2.063	0.0169
FPV(H)⋯GN	T	−2.051	−2.037	0.0141
	Br	—d	—d	—d
	H	−2.053	−2.034	0.0193
FPV(O1/O2)⋯GN	T	−1.904	−1.889	0.0148
	Br	−1.905	−1.890	0.0150
	H	−1.911	−1.896	0.0154
FPV(N/O2)⋯GN	T	—d	—d	—d
	Br	−1.919	−1.904	0.0151
	H	−1.914	−1.896	0.0172
FPV(NH_2_/O1)⋯GN	T	−2.134	−2.122	0.0116
	Br	−2.118	−2.105	0.0123
	H	−2.138	−2.118	0.0206

**Parallel configuration** c				
FPV(R)⋯GN	T	−2.096	−2.084	0.0120
	Br	−2.094	−2.072	0.0214
	H	—d	—d	—d

a All adsorption sites on the surface of the GN sheet are shown in Fig. 2(i).

b *E*_gap_ was assessed according to eqn (2).

c Perpendicular and parallel configurations for all relaxed FPV⋯GN complexes are presented in Fig. S2.

d No adsorption was detected within the designed configuration.

From the data in Table 2, the *E*_HOMO_, *E*_LUMO_, and *E*_gap_ values of the studied systems were denoted with obvious differences before and after the adsorption process. For instance, the *E*_HOMO_ value of the pure GN sheet was −2.356 eV, and was altered to −2.096 and −2.094 eV after the adsorption process within the FPV(R)⋯T@GN and ⋯Br@GN complexes, respectively. Besides, changes in the *E*_gap_ values of the FPV drug and GN sheet were noticed after the adsorption process, indicating the potentiality of the GN sheet to adsorb the FPV drug. For example, *E*_gap_ values of 0.0185 and 0.012 eV were observed in the case of the pure GN sheet and the FPV(R)⋯T@GN complex, remarking the occurrence of the adsorption process. The small *E*_gap_ values also demonstrated the feasibility of transferring the charge within the complex.

### Charge transfer calculations

Bader charge method is one of the most effective tools for assessing the charge transfer over the adsorption process.^54–56^ Therefore, the charge transfer (*Q*_t_) was evaluated for the perpendicular and parallel configurations of the FPV⋯GN complexes at all adsorption sites, and the findings are summarized in Table 1.

Based on the data enrolled in Table 1, all *Q*_t_ values were noticed with a negative sign, revealing that the charges shifted from the FPV drug toward the GN sheet. For FPV⋯GN complexes in perpendicular configuration, *Q*_t_ data after the adsorption process at the H@GN site showed the largest values, followed by Br@GN, then the T@GN sites for almost all the investigated complexes. For example, the *Q*_t_ values of the FPV(F)⋯H@GN, FPV(F)⋯Br@GN, and FPV(F)⋯T@GN complexes were −0.0079, −0.0074, and −0.0070*e*, respectively.

Comparing the results, the adsorption of the FPV drug on the GN sheet in parallel configuration showed more obvious *Q*_t_ values than the perpendicular one, demonstrating the further favourability of the former one. For instance, the *Q*_t_ values of the FPV(R)⋯T@GN and FPV(NH_2_/O1)⋯T@GN complexes were −0.0377 and −0.0221*e*, respectively (Table 1).

**Fig. 4:**
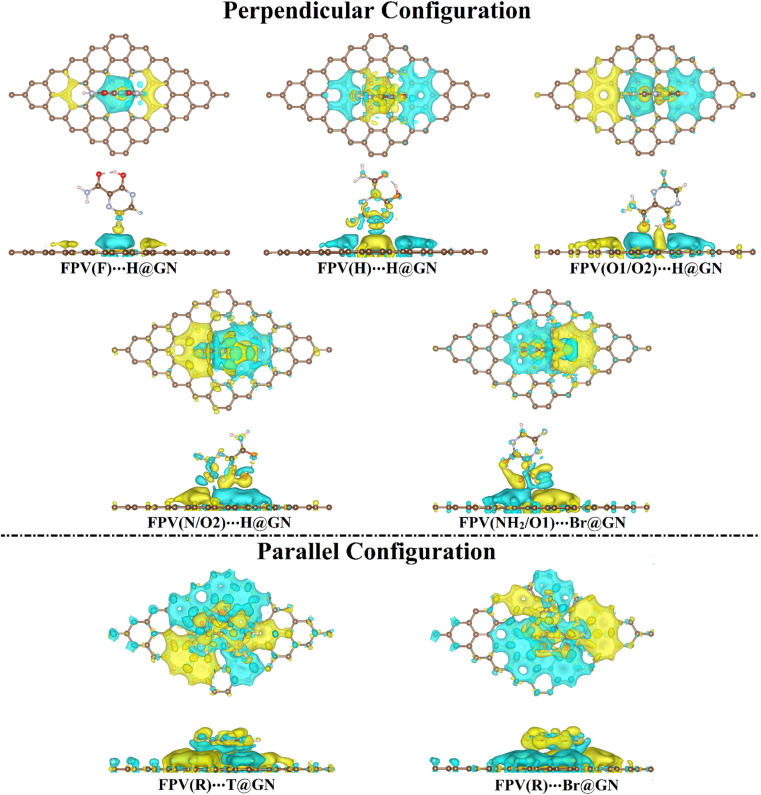
Charge density difference (Δ*ρ*) maps of the most preferred FPV⋯GN complexes in perpendicular and parallel configurations with top and side views. Yellow and cyan colors represent electron accumulation and depletion, respectively. The isosurface value is set to be 7.41 × 10^−5^*e* Å^−3^. Brown, gray, red, green, and pink balls refer to carbon, nitrogen, oxygen, fluorine, and hydrogen atoms, respectively.

To investigate the distribution of charge, the maps of the charge density difference (Δ*ρ*) were generated based on Bader charge analysis for the most preferential FPV⋯GN complexes (*i.e.*, complexes showing the highest negative *E*_ads_ values) and are plotted in Fig. 4.

As illuminated in Fig. 4, yellow and cyan colored regions were conspicuously denoted, outlining the existence of electron accumulation and depletion sites, respectively.

Evidently, the Δ*ρ* maps of the FPV⋯GN complexes in the perpendicular configuration showed that the distribution of the electron accumulated region was compatible with the *E*_ads_ results, revealing the favorability of the H@GN site to adsorb FPV drug (Fig. 4). Further, huge electron accumulated regions were observed in the parallel configuration of the FPV⋯GN complexes more than in the perpendicular one, affirming the further preferentiality of the anterior one.

Bader charge findings were found to be in agreement with the *E*_ads_ results, as listed in Table 1. Among all the complexes under study, the FPV(R)⋯T@GN complex showed the largest negative *Q*_t_ value and the most observable electron accumulated region. Consequently, Bader charge findings disclosed the electron-donating property for the FPV drug in both configurations during the adsorption process on the GN sheet.

### Band structure calculations

To get an overall insight into the adsorption process of the FPV drug on the GN sheet, electronic band structure plots were generated for pure and combined GN sheets (Fig. 2(ii) and 5, respectively).

**Fig. 5:**
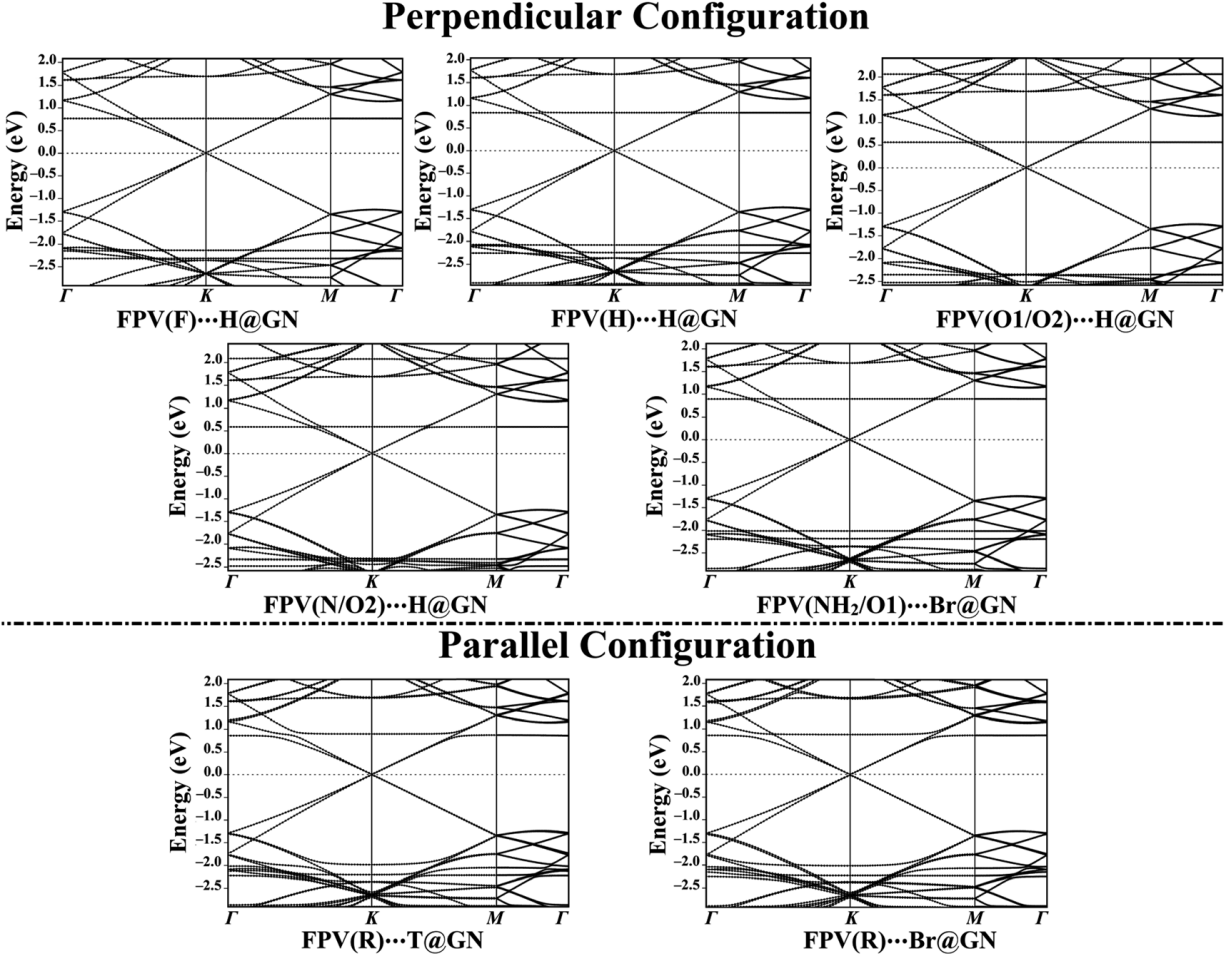
Band structure plots of GN sheet after the adsorption process of the FPV drug at the most preferred sites in perpendicular and parallel configurations. Energies are given relative to the Fermi energy, and the Fermi level is located at zero.

In comparison to Fig. 2(ii) and 5 revealed the notable differences in the band structure plots of GN sheet following the adsorption process of the FPV drug on its surface. According to the data of perpendicular configuration displayed in Fig. 5, new additional bands were observed for the FPV(F)⋯H@GN complex at −2.37 and 0.73 eV in valence and conduction bands, respectively. As well, the bands shifted far away from each other at −2.20 eV, indicating the occurrence of the adsorption process of the FPV drug on the GN sheet. Additionally, new bands were spotted for the FPV(H)⋯H@GN and FPV(NH_2_/O1)⋯H@GN complexes at −2.27 and −2.22 eV in the valence region, while in the conduction region, they were observed at 0.80 and 0.85 eV, respectively.

Similarly, new valence and conduction bands were noticed for FPV(O1/O2)⋯H@GN and FPV(N/O2)⋯H@GN complexes (Fig. 5). Notably, new valence bands in the band structure of FPV(O1/O2)⋯H@GN and FPV(N/O2)⋯H@GN complexes were observed at −2.37/−2.53 and −2.35/−2.48 eV, respectively. At the same time, additional conduction bands for the abovementioned complexes were noticed at 2.05/0.55 and 2.07/0.57 eV, respectively.

For the FPV(R)⋯T@GN and FPV(R)⋯Br@GN complexes, additional valence and conduction bands were noticed at energies around 0.82 eV in the conduction region along with −2.02 and −2.27 eV in the valence region. The appearance of these new bands ensured the occurrence of the adsorption process.

Summing up, the electronic characteristics of the GN surface were changed after combining with the FPV drug in the parallel configuration, which was commensurate with the adsorption energies and Bader charge results (Table 1).

### Density of state (DOS) calculations

**Fig. 6:**
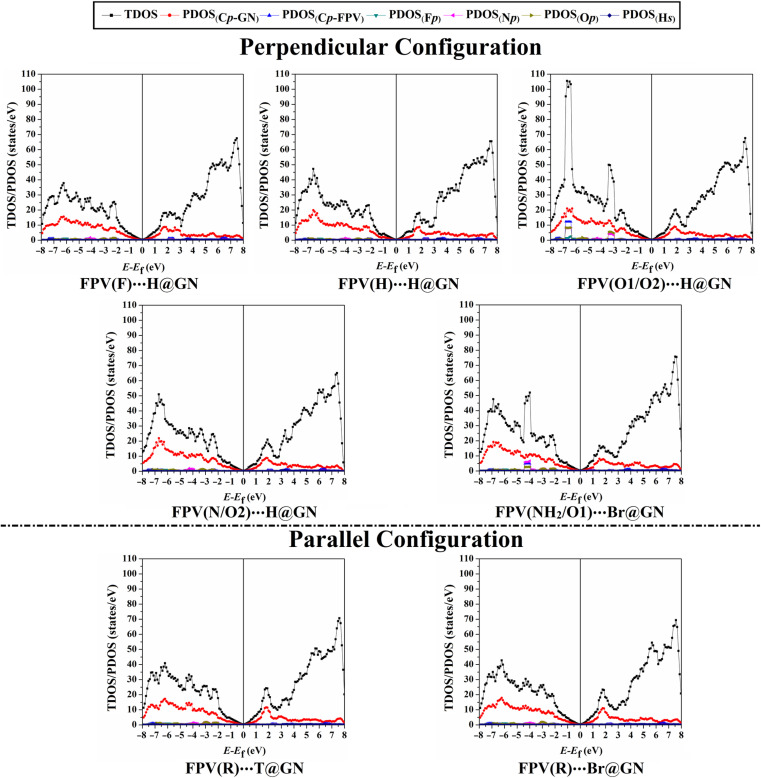
TDOS and PDOS of the most preferred FPV⋯GN complexes in perpendicular and parallel configurations. The contributions of the p-orbital of carbon (C_p_), fluorine (F_p_), nitrogen (N_p_), oxygen (O_p_), and the s-orbital for hydrogen (H_s_) in the adsorption process.

Density of states (DOS) analyses for GN surface before and following adsorption of FPV drug were executed to measure the contributions of each molecular orbital in terms of total and projected DOS (TDOS and PDOS). Fig. 6 displays the TDOS and PDOS for the GN sheet of the FPV⋯GN complexes at the most preferred sites in the perpendicular and parallel configurations. Comparison between Fig. 2(iii) and 6 outlined observable changes for TDOS and PDOS peaks, reflecting the impact of the adsorption process of FPV drug on the surface of the GN sheet.

For the perpendicular configuration, it can be seen that the PDOS peaks of F_p_, N_p_, O_p_, and C_p-FPV_ for FPV⋯GN complexes had a notable contribution to the adsorption process, which was denoted below −2.0 eV in the valence region. While a small contribution of H_s_ was observed, detecting at energy values ranging from 2.7 to 4.5 eV.

For FPV(R)⋯T@GN and FPV(R)⋯Br@GN complexes, the most apparent contributions were noticed for C_p-FPV_, N_p_, F_p_, and O_p_ in the valence region in the range from −2.0 to −7.5 eV. The emergence of these new peaks confirmed that the adsorption process had actually occurred.

To sum up, DOS findings outlined that the adsorption of FPV drug on GN sheets in perpendicular and parallel configurations changed the electronic characteristics of the pure GN surface. The adsorption process had not affected the presence of the Dirac point at the Fermi level, demonstrating physical adsorption of the FPV drug on GN sheet.

### Recovery time

Recovery time (*τ*) is a crucial parameter affecting the drug delivery process and reflects the difficulty of the drug desorption process.^57^ Utilizing eqn (5), *τ* values of the most favorable FPV⋯GN complexes were computed and are collected in Table 3. It is worth mentioning that moderate recovery time is a preferable parameter that addresses the applicability of the utilized nanosheet as a drug delivery system. More detailedly, long and short recovery times led to difficulty in adsorbing and releasing the drug, in conjugation, on and from the surface of the utilized nanosheet.

**Table 3:** Recovery time (*τ*, in ns) of the most favorable FPV⋯GN complexes

System	*τ* (ns)
FPV(F)⋯H@GN	0.37
FPV(H)⋯H@GN	18.46
FPV(O1/O2)⋯H@GN	10.21
FPV(N/O2)⋯H@GN	100.29
FPV(NH_2_/O1)⋯Br@GN	78.60
FPV(R)T@GN	4.72 × 10^8^

According to the listed data in Table 3, the *τ* values of the most desirable FPV⋯GN complexes were 0.37, 18.46, 10.21, 100.29, 78.60, and 4.72 × 10^8^ ns for FPV(F)/(H)/(O1/O2)/(N/O2)⋯H@GN, FPV(NH_2_/O1)⋯Br@GN, and FPV(R)⋯T@GN, respectively. Apparently, the FPV(R)⋯T@GN complex had the longest recovery time of 4.72 × 10^8^ ns, which was in line with its *E*_ads_ value of −15.95 kcal mol^−1^ (Table 1). Remarkably, the obtained moderate values of *τ* ensured the potentiality of the GN sheet for working as a drug delivery system.

## Conclusion

The applicability of the GN sheet as an FPV drug delivery system was herein systematically investigated by means of the adsorption process within the FPV⋯GN complexes in the perpendicular and parallel configurations. Numerous DFT computations, including geometry optimization, adsorption energy, charge transfer, band structure, and DOS, were executed. According to the findings, the GN sheet showed further preferentiality toward adsorbing the FPV drug in the parallel configuration more than in the perpendicular one, which was ensured by a higher negative *E*_ads_ value up to −15.95 kcal mol^−1^. The favorability of the adsorption process in the parallel configuration could be ascribed to the contribution of π–π stacking to the adsorption process within the studied complexes. In the perpendicular configuration, the efficacy of the GN sheet to adsorb FPV drug generally increased in the subsequent order: FPV⋯T@GN < FPV⋯Br@GN < FPV⋯H@GN. Apparently, the FPV(R)⋯T@GN complex had the highest negative *E*_ads_ value of −15.95 kcal mol^−1^ among all the studied complexes in the parallel configuration. From FMO calculations, an alteration in the *E*_HOMO_, *E*_LUMO_, and *E*_gap_ values of the GN sheet was observed, confirming the occurrence of the adsorption process. Bader charge outlines of the FPV⋯GN complexes unveiled the electron-donating and -accepting characters for the FPV drug and the GN sheet, respectively. In line with the adsorption energy pattern, the largest negative *Q*_t_ value along with the most noticed electron accumulated region was observed in the case of the FPV(R)⋯T@GN complex. Upon band structure results, the pure GN sheet had a semiconductor property that was indicated by the presence of the Dirac point, which was not affected after the adsorption of the FPV drug. While observable changes in the PDOS peaks of the C_p-GN_ in pure GN sheet after combining with the FPV drug were detected in the DOSs plots. Such appearance of the new bands and peaks on the band structure and DOSs plots, respectively, outlined the occurrence of the adsorption process. Moderate recovery time values outlined that GN sheet is a potent FPV drug delivery system. These observations would underpin versatile future research relevant to biomedical applications of the GN sheet as a drug delivery system.

## Data availability

The data used in this work can be made available upon reasonable request to the corresponding author.

## Author contributions

Mahmoud A. A. Ibrahim: conceptualization, methodology, software, resources, project administration, supervision, writing—review and editing. Manar H. A. Hamad: data curation, formal analysis, investigation, visualization, writing—original draft. Amna H. M. Mahmoud: methodology, investigation, project administration, writing—review and editing. Gamal A. H. Mekhemer: supervision, writing—review and editing. Peter A. Sidhom: writing—review and editing. Shaban R. M. Sayed: resources, writing—review and editing. Nayra A. M. Moussa: project administration, writing—review and editing. Abdallah I. M. Rabee: writing—review and editing. Eslam Dabbish: writing—review and editing. Tamer Shoeib: conceptualization, resources, writing—review and editing.

## Conflicts of interest

The authors declare that they have no conflicts of interest.

## Supplementary Material

RA-013-D3RA03227B-s001
