# Supplementary material for: Adsorption of Favipiravir on pristine graphene nanosheets as a drug delivery system: a DFT study
Source: RSC Adv. 2023 Jun 9;13(26):17465–75. doi: 10.1039/d3ra03227b (PMC10253565; doi:10.1039/d3ra03227b)
Supplement: RA-013-D3RA03227B-s001 [file RA-013-D3RA03227B-s001.pdf]

Electronic Supplementary Material (ESI) for RSC Advances.
This journal is © The Royal Society of Chemistry 2023

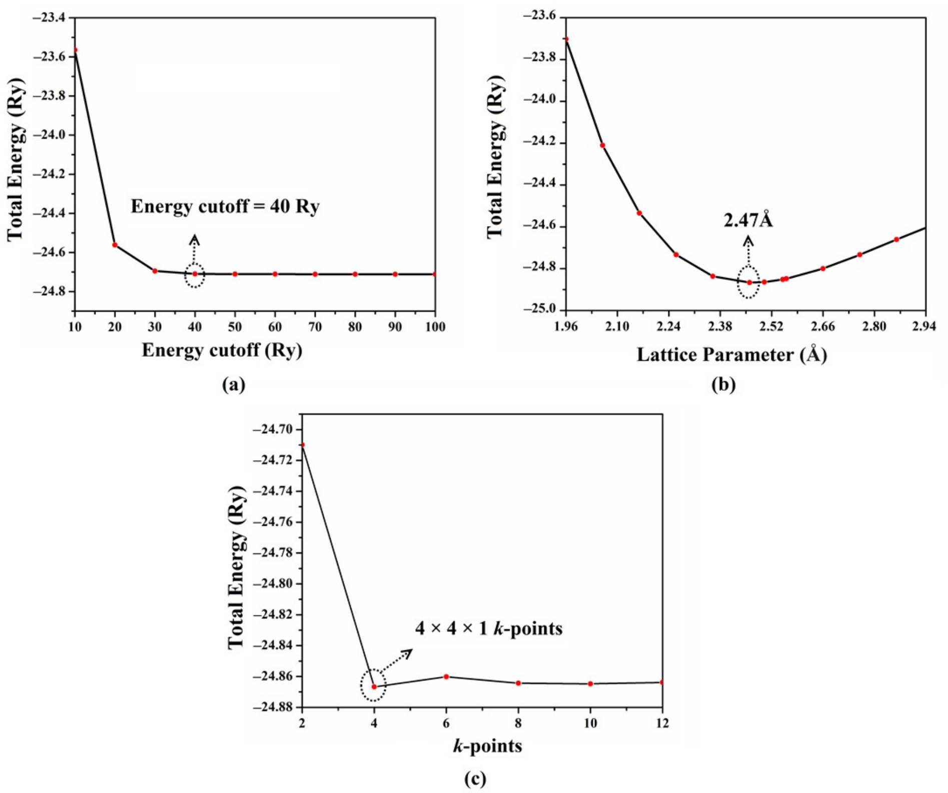

Figure S1. Convergence tests plots of the total energy with the (a) cutoff energy, (b) lattice parameter, and (c) *k*-point of the unit cell of GN with two C-atoms.

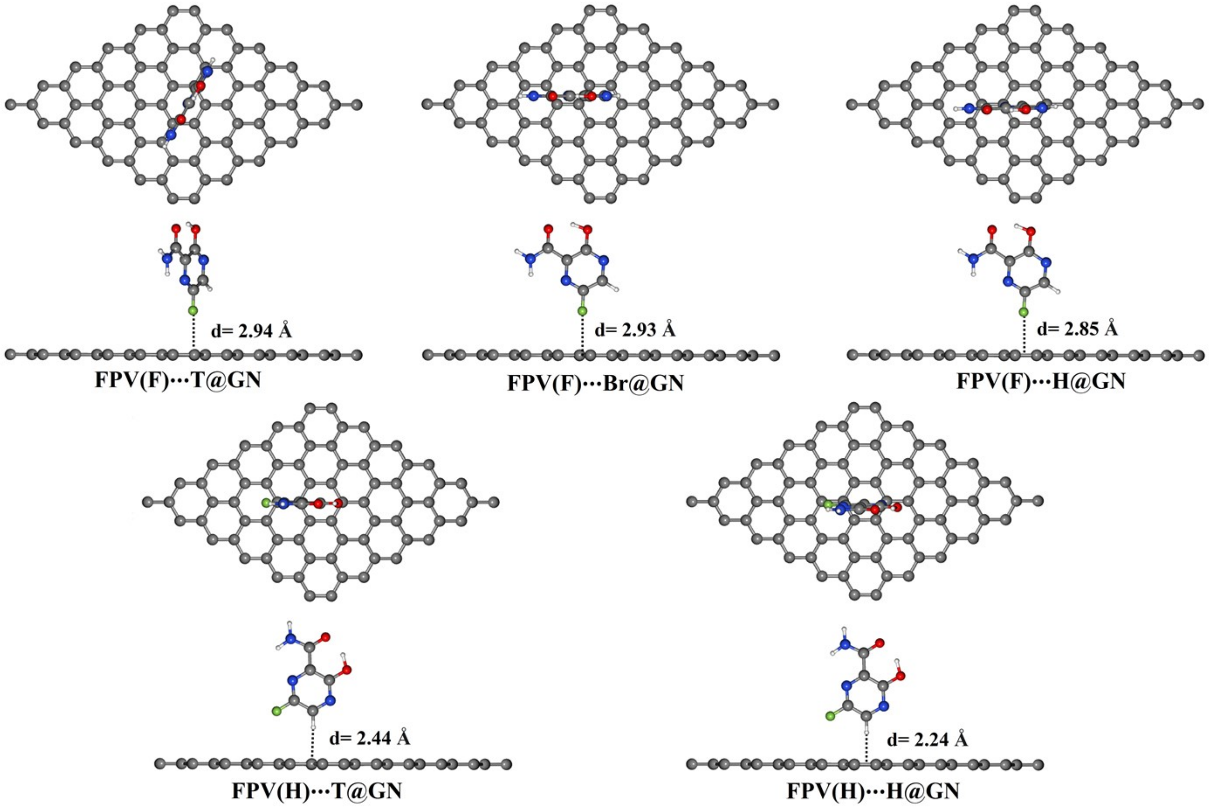

Figure S2. Relaxed structures of all FPV···GN complexes in perpendicular and parallel configurations with top and side views. The distance (d) between FPV drug and GN sheet are provided in Å.

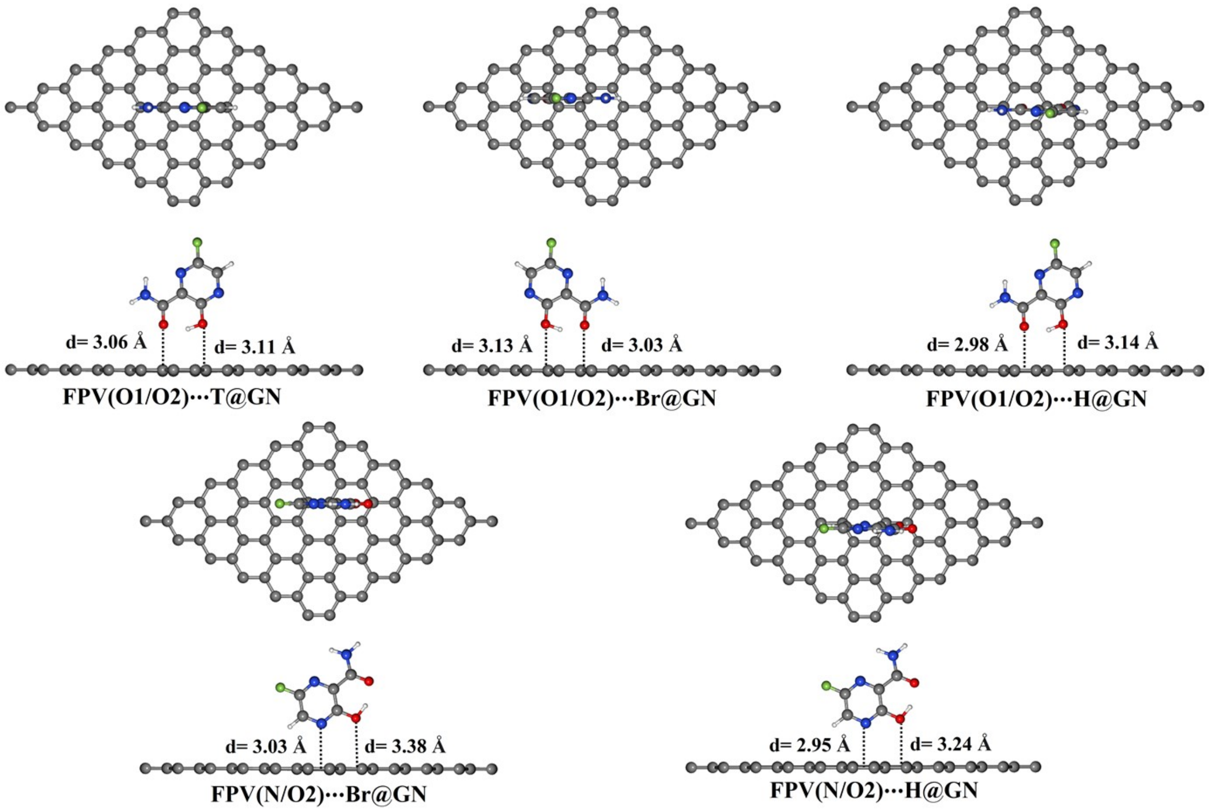

Figure S2. Continue.

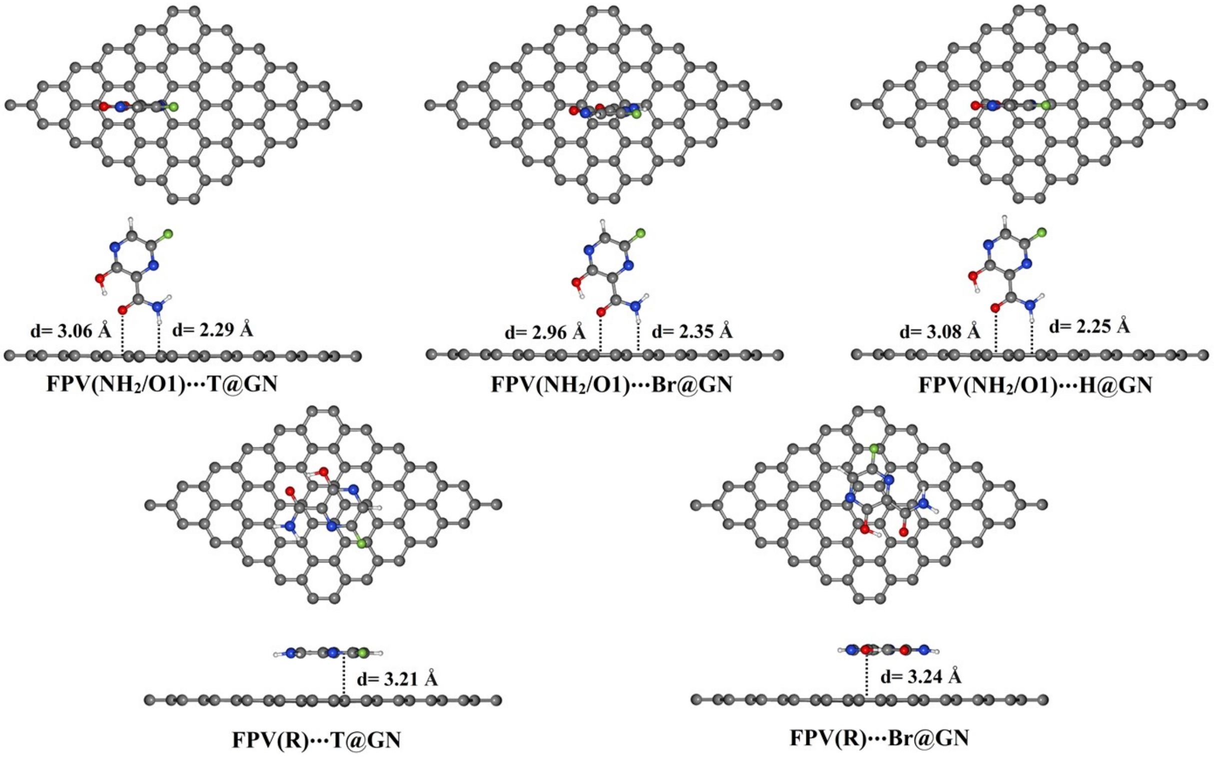

Figure S2. Continue.
